# PP21 Examining Clinical Evidence: Incorporation Of Medications For Ultra-Rare Diseases – Descriptive Analysis Of Conitec Reports (2012 to 2022) – Brazil

**DOI:** 10.1017/S0266462324001946

**Published:** 2025-01-07

**Authors:** Juliana Machado-Rugolo, Daniel da Silva Pereira Curado, Denis Satoshi Komoda, Luis Gustavo Modelli de Andrade, Mônica Aparecida de Paula De Sordi, Alan Francisco Fonseca, Juliana Tereza Coneglian de Almeida, Silvana Molina Lima, Silke Anna Theresa Weber, Marisa Santos, Jean-Eric Tarride, Lehana Thabane, Marilia Mastrocolla de Almeida Cardoso

## Abstract

**Introduction:**

The decision-making process for health technology assessment (HTA) in ultra-rare diseases faces a significant challenge for agencies worldwide. This study sought to offer an analytical overview of the clinical evidence outlined in the recommendations of the Brazilian National Committee for Health Technology Incorporation (Conitec) in ultra-rare diseases.

**Methods:**

Data were extracted from recommendation reports for the ultra-rare diseases evaluated between 2012 and 2022. To classify a disease as ultra-rare, the epidemiological criterion or a consultation with the Orphanet platform was used (prevalence of ≤1/50,000 inhabitants). The extracted variables included the type of evidence synthesis, type of studies, instrument, the result of the assessment of the methodological quality of the studies, the format of evidence synthesis presentation, whether the evidence was graded, and the result.

**Results:**

Among 53 analyzed reports, 70 percent relied on randomized controlled trials, followed by systematic reviews (SR), and observational studies. Reports with positive recommendations based on SR comprised 63 percent. GRADE applied to 27 reports and indicated low or very low results for the first two outcomes (62% and 65%). No clear link between evidence quality and final recommendations was observed. Meta-analysis-based reports had 83 percent positive recommendation rate, compared to 55 percent without meta-analysis. Surrogate outcomes were predominant. Clinical characteristics significantly influenced final decisions, especially when new data emerged in public consultation or had the potential to alter disease progression, reduce severe events, or enhance survival.

**Conclusions:**

Ultra-rare diseases pose challenges in evidence quantity and quality. Traditional HTA frameworks seem inadequate, lacking robust evidence for these conditions. The difficulties in ultra-rare disease HTA underscore the need for specialized frameworks. This analysis acknowledges limitations, notably the heterogeneity in older report structures compared to recent ones, reflecting evolving HTA methodologies in Brazil.

